# A regulatable transgene expression system for cultured *Plasmodium falciparum *parasites

**DOI:** 10.1186/1475-2875-7-86

**Published:** 2008-05-20

**Authors:** Christian Epp, Dima Raskolnikov, Kirk W Deitsch

**Affiliations:** 1 Department of Microbiology and Immunology, Weill Medical College of Cornell University, 1300 York Avenue, W-704, Box 62, New York, NY 10021, USA; 2 Department of Parasitology, Heidelberg University Medical School, Im Neuenheimer Feld 324, 69120 Heidelberg, Germany

## Abstract

**Background:**

The ability to transfect and create transgenic cultured malaria parasites has transformed the study of *Plasmodium falciparum *over the last decade. With the completion of the annotated genome sequence, the process of gene discovery now routinely includes gene knockouts, over-expression and complementation analysis. However, while this technology has proven extremely valuable, significant limitations exist. In particular, *P. falciparum *DNA is often unstable and difficult to clone because of its AT-rich, repetitive nature. As a result, transgene expression constructs can be difficult to assemble due to the need to include two expression cassettes on a single plasmid, one to drive expression of the transgene of interest and a second for expression of the selectable marker. In addition, transgene expression levels are usually not regulatable, making it difficult to assess phenotypes that are sensitive to the amount of protein expressed.

**Results:**

A plasmid based system for transgene expression is described that uses a single, bidirectional promoter to drive expression of both the transgene and the selectable marker, thus greatly reducing the size of the construct and enhancing stability. Further, by altering the concentration of drug used for selection, it is possible to modulate the copy number of the concatameric episomes and thereby regulate the expression level of the transgene through a range greater than 10 fold.

**Conclusion:**

The transgene expression system described here should prove useful for both routine protein over-expression and complementation experiments as well as for experiments in which precisely manipulating the expression level of candidate proteins is desirable. This should provide an additional level of precision to the tools used to study the molecular biology of malaria parasites.

## Background

Over a decade ago, the first transfections and genetic manipulations of malaria parasites were accomplished [1-3], ushering in a new era in the study of the molecular biology and biochemistry of both human and rodent parasites. Since then, numerous studies have been published utilizing transfection to both knockout various genes as well as to over-express modified transgenes to investigate such aspects of parasite biology as mechanisms of drug resistance and protein trafficking[4,5]. Combined with the release of the annotated genome sequences of numerous parasite species as well as several different isolates of *Plasmodium falciparum*, there have been unprecedented recent advances in gene discovery in malaria parasites[6]. Thus the development of the current tools used to genetically manipulate parasites continues to contribute substantially to the understanding of the basic biology of malaria.

While the ability to genetically manipulate cultured *P. falciparum *parasites has proven to be extremely useful, the tools that are currently used have several limitations. For example, all systems for transfecting cultured parasites are based on circular plasmids that are initially created and produced using transformed *Escherichia coli*. These plasmids are then introducing into cultured parasites. The requirement that all constructs must first transition through *E. coli *necessitates that the parasite DNA sequences included on the plasmids be stably replicated by the bacteria, a property that many sequences lack due to the extremely AT-rich, repetitive nature of the *P. falciparum *genome, particularly in non-coding regions[7]. This difficulty is especially pronounced when trying to over-express transgenes from episomal constructs which require two separate cassettes, the first containing the transgene to be expressed, the second containing the selectable marker, and both requiring separate promoter and terminator elements. Such constructs must, therefore, contain several lengthy stretches of *P. falciparum *DNA and stability in *E. coli *can be a significant issue.

A second limitation for parasite transgene expression regards the inability to control and manipulate the expression level of the gene of interest. Currently, the level of expression is a result of the copy number of the episomal plasmid as well as the strength of the promoter driving transcription of the transgene. These two parameters are determined by the DNA sequences included in the plasmid and therefore can only be changed by making substantial alterations to the construct and repeating the transfection. The ability to regulate expression levels directly within transfected parasites would provide an additional layer of control and allow researchers to investigate phenotypes resulting from different amounts of expressed protein. Recently, several systems have been described which allow the regulation of gene expression, e.g. via Atc-inducible system[8], the use of a protein destabilization domain[9] or ribozymes[10]. However, due to the complexity of these systems their utility for widespread use in malaria research remains to be proven[11].

A new system for transgene expression is described here that addresses some of the limitations mentioned above. By utilizing DNA sequences that contain bidirectional promoter activity, expression constructs were created that use a single stretch of *P. falciparum *intergenic sequence to drive expression of both a transgene as well as a selectable marker, resulting in a smaller, more stable plasmid. Second, by using the *blasticin-S-deaminase *selectable marker and altering the concentration of blasticidin used for selection, it is possible to tightly control and manipulate the copy number of episomes carried by the parasites. In turn this leads to controlled levels of transgene expression, thus providing a novel manipulatable expression system for cultured *P. falciparum *parasites. This system should prove valuable for studies that employ over-expression to determine protein function or localization, as well as in complementation experiments used to validate knockout phenotypes.

## Methods

### *Plasmodium falciparum *culture and transfection

All experiments utilized the *P. falciparum *NF54 line cultivated at 5% haematocrit in RPMI 1640 medium, 0.5% Albumax II (Invitrogen, Carlsbad, California, United States), 0.25% sodium bicarbonate, and 0.1 mg/ml gentamicin. Parasites were incubated at 37°C in an atmosphere of 5% oxygen, 5% carbon dioxide, and 90% nitrogen. Parasites were transfected by using "DNA loaded" red blood cells as previously described[12]. Briefly, 0.2 cm electroporation cuvettes were loaded with 0.175 ml of erythrocytes and 50 μg of plasmid DNA in incomplete cytomix solution. Electroporation conditions were 0.31 kV and 960 mFD. For stable transfections, parasites were cultured in media containing either 40 ng/ml WR99210 or the designated concentration of blasticidin.

### DNA constructs

Construction of pHLIDH constructs: Plasmids pVLH and pVLHIDH were described previously[13]. The luciferase coding region and *hrp2 *3'UTR of pVLH were amplified by PCR introducing restriction sites for *Pst*I (5') and *Kpn*I (3') using the primers *FflucPstI*: 5'-AACTGCAGGCATGGAAGACGCCAAAAAC-3' and *hrp3'downKpnI*: 5'-GGGGTACCCGCCTCTCCCCGCGCGTTGG-3'. Subsequently, this fragment was ligated into *Pst*I/*Kpn*I digested pVLHIDH to replace the VLH portion (*var *promoter, *luciferase *and *hrp2 *3'UTR) to yield plasmid pHLIDH. In the resulting plasmid, the PFB1055c intron acts as a promoter for both the reporter and drug resistance genes. Additional intron sequences were first amplified from genomic DNA with the following primers, thereby adding the restriction sites *BamH*I and *Sal*I to the 5' and 3'ends, respectively, and inserted into *BamH*I/*Sal*I digested pHLIRH. Gene specific primers for *var *introns: PFC0005w: 5'-CGGGATCCGAAGGTAAAAGAGAATATATATGTG-3'/5'-GCGTCGACTTCTAAAATAATAAAAGAGG-3'; PFF0845c: 5'-CGGGATCCCTAAAGGTATTATATATG-3'/5'-GCGTCGACGATCAATAGTAGATTTGG-3'; PFD0020c: 5'-CGGGATCCGCGCTTTTATTTTTGAAGG-3'/5'-GCGTCGACGGTCCACAGGAGATTTAGG-3'. For the construction of pHLsti/CamDH, the intron in pHLIDH (*Sma*I and *Sal*I) was replaced with the PfCam promoter from pHH-Tati2 construct (*Hpa*I and *Sal*I) (gift of M. Meissner)[8].

Contruction of pHBIRH vectors: Luciferase gene was cut out pHLIRH with *Hind*III and *Sma*I and replaced with the blasticidin S deaminase gene obtained by restriction digestion of pVcBB/IDH[14] with *Hind*III and *Hpa*I. Restriction sites *BamH*I and *Sal*I were used to exchange bidirectional promoters between plasmids pHLIDH, pHLIRH and pHBIRH.

### *Renilla *and firefly *luciferase *assays

Parasites were synchronized by magnetic cell separation using MACS CS columns (Miltenyi Biotech) and confirmed by light microscopy. Parasitaemias were counted for 1000 RBCs. For luciferase assays, parasites were obtained from 200 μl of culture by centrifugation and subsequent lysis in 100 μl of Glo Lysis Buffer (Promega). To determine firefly luciferase expression, 100 μl of Bright-Glo Luciferase reagent was added to the lysate and luminescence was measured immediately in a TD-20/20 luminometer. For *Renilla *luciferase expression, 100 μl *Renilla *assay reagent (Promega) was added to the lysate and luminescence determined using the TD-20/20 luminometer. For both *Renilla *and firefly luciferase, luminescence was expressed per 1% parasitaemia.

### Quantitative Realtime PCR assays

RNA was extracted from synchronized ring stage parasites 16–18 h or late trophozoites 40–42 h post-invasion. RNA extraction was performed with the TRIZOL LS Reagent (Invitrogen) as previously described[15]. RNA to be used for cDNA synthesis was purified on PureLink column (Invitrogen) according to manufacturer's protocol. Isolated RNA was then treated with Deoxyribonuclease I (Invitrogen) to degrade contaminating gDNA. cDNA synthesis was performed with Superscript II Rnase H reverse transcriptase (Invitrogen) as described by the manufacturer. cDNA was synthesized from 800 ng total RNA in a reaction volume of 20 μl. For each cDNA synthesis reaction, a control reaction without reverse transcriptase was performed with identical amounts of template and primers. Realtime PCR was carried out as described previously[14]. All reactions were performed at a final primer concentration of 0.5 μM using Bio-Rad ITAQ SYBR SUPERMIX^® ^in 20 μl reactions on an ABI Prism^® ^7900HT real-time PCR machine.

All reactions included primers for the control genes seryl-tRNA synthetase (PF07_0073), fructose biphosphate aldolase (PF14_0425) and actin (PFL2215w), as described by Salanti et al[16], as well as arginyl-tRNA synthetase (PFL0900c) and glutaminyl-tRNA synthetase (PF13_0170), as described by Dzikowski et al[14]. The ΔCT for each individual primer pair was determined by substracting the individual CT value from the CT value of the control the *seryl-tRNA *synthetase. ΔCTs were then converted to relative copy numbers with the formula 2^ΔCt^. Primers for luciferase[17] and blasticidin[14] were previously described. Additional gene-specific primer pairs were designed using primer 3 software[18]: *Renilla luciferase*: 5'-TTCGAAAGTTTATGATCCAG-3'/5'-AACATGTCGCCATAAATAAG-3'

*msp*1: 5'-TACAAGTCCATCATCTCGTT-3'/5'-TGGTTAAATCAAAGAGTTCG-3'

*kahrp*: 5'-CAATTACAACCTCAACAACC-3'/5'-TTTTACCATCGACAACATTT-3'

*sbp*1: 5'-AATCCACAACTGATTTGGTA-3'/5'-GAATAGGGGACATAGATTCG-3'.

## Results

### Episomal expression of *luciferase *and *dhfr *in stably transformed parasites

**Figure 1:**
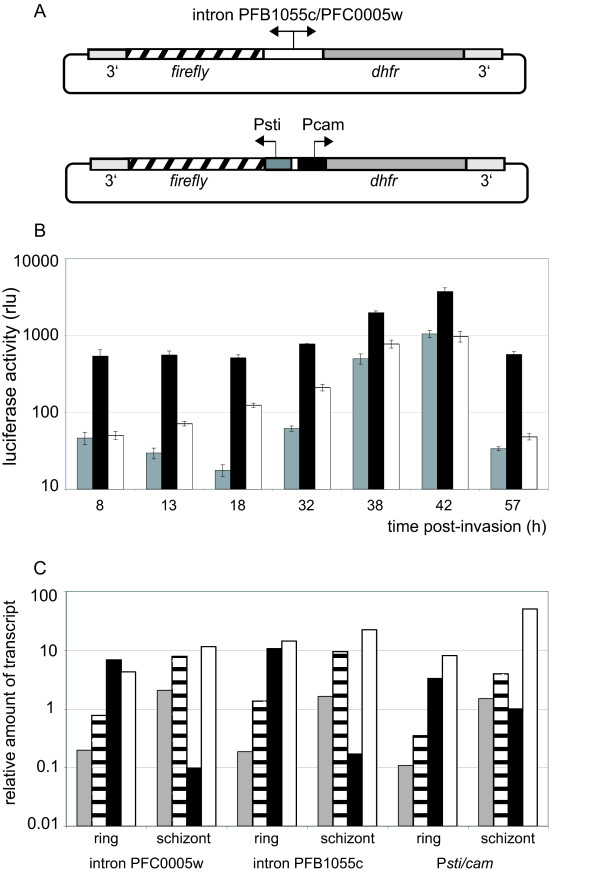
**Episomal expression of *luciferase *with constructs utilizing bidirectional promoters**. A: Diagram of bidirectional constructs: introns of *var *genes PFB1055c and PFC0005w are shown in the top diagram while the 5' regions of *calmodulin *gene (*Pcam*) and the stress-inducible gene (*Psti*) are shown on the bottom. *firefly *refers to the *firefly luciferase *gene. 3'denotes the 3'untranslated region of the *Pfhrp2 *gene. The total construct lengths are 7162 bp, 7254 bp and 7315 bp for plasmids containing the PFB1055c, PFC0005w and *Pcam/Psti *promoters, respectively. B: *P. falciparum *NF54 stably transformed with bidirectional constructs containing *var *intron PFB1055c (gray), *var *intron PFC0005w (black) and *Psti/Pcam *(white) promoters were synchronized by magnetic cell separation and luciferase activity was measured at different time points throughout the asexual cycle. Samples were measured in triplicates and error bars indicate the standard deviation. C: mRNA levels were determined by synthesis of cDNA followed by quantitative realtime PCR analysis. RNA levels of *dhfr *(dark gray) and *luciferase *(hatched) were compared to *P. falciparum *genes *sbp1 *(black) and *msp1 *(white). RNA was extracted ~16 h (ring) and 40 h (schizont) post-invasion.

In an attempt to create smaller, more stable expression constructs for transfecting cultured *P. falciparum *parasites, plasmids were constructed that contained both the *dhfr *and *luciferase *coding regions arranged such that expression of both genes was driven simultaneously from a single bidirectional promoter (Figure 1A). Three different promoters were compared: two *var *introns from genes PFB1055c and PFC0005w, respectively, and the 5' upstream region of the *Pfcam *gene. The promoter activity of *var *introns has been previously documented[13], and the ~1 kb region upstream of *Pfcam *had been previously shown to have bidirectional promoter activity[3]. In a more detailed analysis[19] it was shown that this sequence is located between two genes in head-to-head orientation and contains two different promoters, one for the *Pfcam *gene and a second one in the reverse orientation for an open reading frame that shows homology to stress-inducible genes in yeast and hence has been named *P. falciparum *stress-inducible gene (*Pfsti*). *P. falciparum *parasites were transfected with all three constructs and, after selection with WR99210, stably transformed parasite lines were obtained with each of the constructs, indicating the *dhfr *gene was expressed at sufficient levels to readily produce drug resistant parasites.

To determine the efficiency of transgene expression from the different constructs, the three parasite lines were synchronized and *luciferase *and *dhfr *expression were analysed at different stages of the intraerythrocytic cell cycle (Figure 1). The construct with *var *intron PFC0005w produced the highest level of *luciferase *expression, although all three transformed lines were well above background. *luciferase *expression of all three parasite lines was significantly higher in late stages than in ring stages (Figure 1B). For the two intron promoters this was expected and corresponds to the previously reported activity of *var *intron promoters and synthesis of non-coding *var *RNAs[20]. The *Psti/cam *promoter is active in a more constitutive manner and shows a lower degree of stage specificity. Compared to *var *intron PFB1055c, the *Psti/cam *promoter becomes active earlier in the cell cycle with luciferase levels ~10 fold higher in ring stage. Its activity shows a slower increase over the course of the cell cycle to finally achieve maximum expression in schizonts with luciferase activity similar to that produced by the construct with the PFB1055c *var *intron.

To quantitatively analyse expression of the *dhfr *gene in the transfected lines, realtime RT-PCR was used to measure steady state mRNA levels. This analysis showed that *dhfr *and *luciferase *expression correlated in all constructs, consistently showing stronger expression in late stages (Figure 1C). This indicates that the promoter activities in the forward and reverse orientations are not regulated independently, but are strictly linked. This is particularly interesting for the promoter region of *Psti*/*cam *as it contains two promoters for different genes that might have been expected to be regulated separately. It has been hypothesized that the *Psti *upstream regulatory region does not contain a constitutive promoter, but rather might be inducible only in response to cellular stress[19]. An induction of *Psti *promoter activity was not observed under temperature stress (data not shown) and it is assumed that the promoter activity measured in this assay corresponds to the "normal" promoter activity of the *Psti *gene. However, the possibility that the *Psti *promoter is inducible under different conditions and that in this assay a low "stand-by" activity was measured cannot be excluded.

Transcription levels of *luciferase *and *dhfr *were compared to two single copy *P. falciparum *genes, skeleton binding protein 1 *(sbp1) *and merozoite surface protein 1 *(msp1)*. The *sbp1 *gene is expressed early in the cell cycle, therefore, *sbp1 *RNA levels in ring stages are much higher than for *luciferase*, while in late stages, *sbp1 *expression is low compared to *luciferase*. Thus the expression of *luciferase *and *sbp1 *are inversely correlated. As a consequence the expression constructs described here would not be expected to be suitable for the expression of ring-specific genes. In contrast, *msp1 *is expressed later in the cell cycle. Although *msp1 *transcription is not exclusively restricted to trophozoites, but starts already in late ring stages, *msp1 *expression increases over the course of the cell cycle and RNA levels peak in late trophozoites[21,22]. Due to the late-stage specific activity of the intron promoters, the expression pattern of *luciferase *corresponds more closely to that of *msp-1*. In schizont stage the expression levels of *luciferase *produced by the construct with the stronger intron promoter (*var *PFC0005w) are very similar to those of *msp1*. Therefore, the pHLIDH constructs might be suitable vectors for the expression of late stage genes such as *msp1*.

### Episomal co-expression of *luciferase *and *blasticidin-S-deaminase *in stably transformed parasites

**Figure 2:**
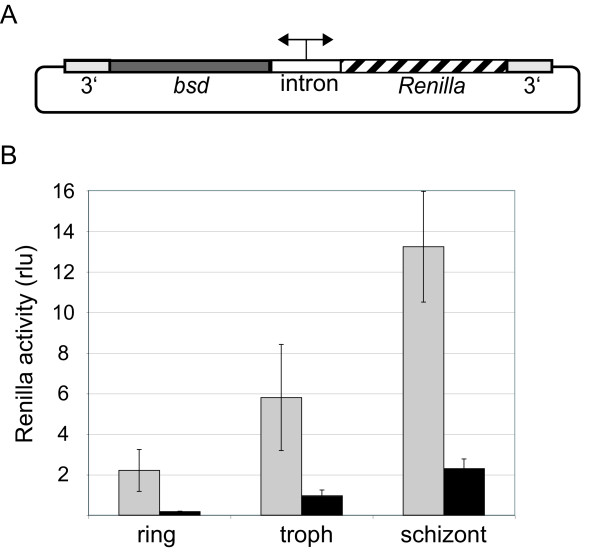
**Cell cycle dependent episomal expression of *Renilla *luciferase from HBIRH with two different *var *intron promoters**. A: Diagram of bidirectional expression construct pHBIRH. The construct contains the intron from either PFC0005w or PFD0020c, resulting in total plasmid sizes of 6380 bp or 6432 bp, respectively. *bsd*: blasticidin-S-deaminase gene; 3': 3'untranslated region of *Pfhrp2*; *Renilla*: *Renilla luciferase *gene; intron: intron *var *gene containing bi-directional promoter activity. B: Luciferase activity was measured from *P. falciparum *NF54 parasites stably transformed with pHBIRH containing either *var *intron PFC0005w (grey) or *var *intron PFD0020c (black) as a bidirectional promoter. Samples were taken at different time points throughout intra-eryhtrocytic cycle: 16 h (ring), 32 h (troph) and 40 hours (schizont) post-invasion. Samples were measured in triplicate and error bars indicate the standard deviation.

In stably transformed *P. falciparum *parasites, episomes are usually present as concatemers[23,24]. This potentially enhances the expression of the resistance marker by providing transfected parasites with multiple copies of the resistance gene. It had previously been observed that episome copy number can vary when blasticidin-S-deaminase (*bsd*) was used as a selection marker, showing a direct correlation of drug concentration and the number of plasmid copies. In the expression constructs described here containing bidirectional *var *introns, expression of the resistance and reporter genes were strictly linked. It was therefore hypothesized that it might be possible to regulate expression levels of a reporter gene (or a transgene of interest) by varying blasticidin concentration in the culture media. For this purpose, constructs were made that contained *bsd *as a resistance marker and *Renilla luciferase *(pHBIRH) as reporter gene, both simultaneously expressed from a bidirectional promoter. Two different *var *introns, PFC0005w and PFD0020c, were used as promoters (Figure 2). These constructs were used to stably transform *P. falciparum *NF54 parasites using selection with 2 μg/ml blasticidin. Both parasite lines displayed the expected stage-specific expression pattern, with approximately five-fold higher *Renilla *luciferase expression in schizonts than in rings. pHBIRH with the PFC0005w intron produced much stronger *Renilla *luciferase expression than did the intron from PFD0020c. Therefore, pHBIRH containing *var *intron PFC0005w was used for further experiments.

### Regulation of *Renilla *luciferase expression by altering blasticidin concentration

**Figure 3:**
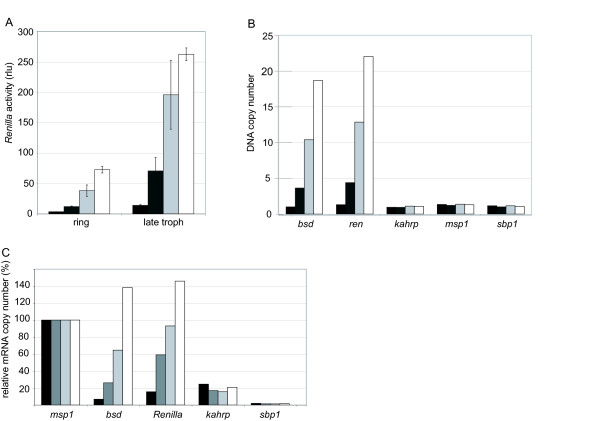
**Regulated expression of *Renilla *luciferase using variable blasticidin selection**. *P. falciparum *NF54 parasites stably transformed with pHBIRH containing *var *intron PFC0005w were grown in presence of different blasticidin concentrations; 2 μg/ml (black), 5 μg/ml (dark grey), 10 μg/ml (light grey) and 20 μg/ml (white). A: *Renilla *luciferase activity measured 16–18 h (ring) and 40–42 h (late troph) after invasion. B: DNA was extracted from parasite cultures and the copy numbers of blasticidin (*bsd*), *Renilla luciferase *(*ren*) and the *Plasmodium *genes *kahrp*, *sbp1 *and *msp1 *were determined by quantitative realtime PCR. Values were standardized to the control gene seryl-tRNA synthetase. Samples were measured in triplicate and error bars indicate the standard deviation. C: mRNA levels were determined by quantitative realtime PCR analysis of cDNA. RNA was extracted from synchronized parasite cultures in the late trophozoite stage. ct values were standardized to control gene seryl-tRNA synthetase and relative mRNA copy numbers are displayed as percentile of *msp1 *(100%).

*P. falciparum *parasites stably carrying pHBIRH (intron PFC0005w) were divided into identical cultures and grown in the presence of 2, 5, 10 and 20 μg/ml blasticidin for four weeks. In the cultures containing 10 and 20 μg/ml blasticidin, dead parasites were detected initially and growth of the cultures slowed down significantly. However, after five cycles (10 μg/ml) or eight to 10 cycles (20 μg/ml), cultures had recovered and parasite growth resumed at usual rates. All four cultures were synchronized and *Renilla *luciferase activity was measured at the ring stage (~16 h post invasion) and trophozoite stage (~40 h post invasion). As expected, *Renilla *luciferase expression was stage-specific with ~five-fold greater activity in trophozoites (Figure 3A). In both stages, a linear correlation of *Renilla *luciferase activity and blasticidin concentration was observed, demonstrating that regulation of an episomally expressed reporter gene is possible by varying the concentration of the drug used for selection.

To investigate if the increase in *Renilla *luciferase expression resulted from an increase in promoter activity or rather reflected an increase in the copy number of the expression construct, DNA was extracted from the different parasite lines and the copy numbers of the *bsd *and *Renilla luciferase *genes were determined by quantitative PCR and compared to several single-copy genes of *P. falciparum*. There was a 10 to 20-fold difference in copy numbers between cultures grown in the presence of 2 μg/ml and 20 μg/ml blasticidin. In all four cultures, the copy numbers of the expression constructs displayed a linear correlation with both blasticidin concentration and *Renilla *luciferase activity (Figure 3B), indicating that the increase in *bsd *and *Renilla luciferase *expression was not due to changes in promoter activity but rather to a change in copy number of the expression construct. Thus the plasmid copy number can be regulated over a 10-fold range by altering the drug concentration used for selection and in this way the expression of the transgene (in this case *Renilla luciferase*) can be co-regulated. Episomal plasmids are usually present as concatemeres of up to 15 copies[23,24] suggesting that changes in concatemer size are the most likely mechanism for copy number variation, however, it is also possible that an increase in copy number is caused by presence of multiple episomes per parasite.

### Manipulating episome copy number to "fine-tune" transgene expression levels

A possible application of these constructs could be the expression of a gene of interest in place of *Renilla luciferase *to investigate its putative biological function. As the effect of over-expressing genes in *Plasmodium *depends on the timing and the strength of expression[25], the expression levels of *Renilla luciferase *and *bsd *were compared to the expression levels of several endogenous *P. falciparum *genes. cDNA was prepared from trophozoites and subsequently analysed by quantitative PCR. As expression from intron promoters is restricted to late stages, we compared the expression levels to that of *msp1*, a gene that is similarly primarily expressed in late stages. The relative amount of *bsd *and *Renilla luciferase *RNA directly correlated with blasticidin concentration, corroborating the results of the *Renilla luciferase *activity measurement (Figure 3C). RNA levels of *Renilla luciferase *were lower than *msp1 *for blasticidin concentrations of 2 and 5 μg/ml, however parasites growing in the presence of 10 μg/ml showed nearly equal expression levels of *Renilla luciferase *and *msp-1 *(*Renilla luciferase *92% of *msp1*). In parasites growing in the presence of 20 μg/ml, *Renilla luciferase *RNA levels were ~40% higher than *msp1 *RNA. Other genes that are ring-stage specific (*kahrp *and *sbp1*) were expressed at lower levels in late trophozoites and served as controls. These results show that with the novel expression constructs described here, gene expression can be regulated over an approximately 10-fold range and, as was exemplified with *msp1*, expression levels can be achieved that correspond to endogenous transcription levels. In addition, the effect of a co-expressed gene (or a truncated version thereof) can be analysed by choosing concentrations that result in mRNA amounts above or below the "normal" expression level.

## Discussion

The regulatable expression system described here appears to rely on the flexibility in copy number of episomal plasmid concatamers. It, therefore, seems unlikely that this system will work with constructs that have integrated into the parasite's genome. It is probably best that experiments in which alterations in transgene expression are desired be performed on parasites shortly after stably transformed lines have been obtained in order to avoid the chromosomal integrations that are typical after long-term culture of transfected parasites. In addition, the plasmid constructs described here all utilize bidirectional promoters that are primarily active late in the cell cycle. In theory, similar plasmids could be constructed that utilize a ring stage specific promoter and thus allow regulated expression early in the cell cycle. A similar system was previously described that utilized the constitutively active bi-directional promoter of the *ef-1α *gene of *Plasmodium berghei*, which might provide expression through a broader range of the parasite cell cycle[26].

The blasticidin resistance gene is the ideal selectable marker for the type of manipulatable system described here. Unlike the *dhfr *selectable markers, which encode proteins that are resistant to the drug used for selection, *bsd *instead encodes a protein that inactivates blasticidin. Thus, the addition of increasing amounts of blasticidin requires corresponding increases in blasticidin-S-deaminase encoded by *bsd *in order to detoxify the compound and maintain parasite viability. By driving expression of the transgene of interest with the same promoter that drives *bsd *expression, increased *bsd *expression necessitates increased transgene expression, giving the regulatable expression that is desired. Removal of blasticidin from the media results in rapid loss of the transfecting plasmid (data not shown), suggesting that transgene expression levels can be lowered by reducing blasticidin selection pressure. In addition, since blasticidin is not an anti-malaria drug commonly used in the field, all parasite isolates typically used for molecular genetic studies are sensitive to this drug, and therefore the constructs described here should work in all of these laboratory lines. It is not clear if this type of system will work with rodent parasites like *Plasmodium berghei*. These parasites must be reared in their mammalian hosts, and the effect of blasticidin on host viability has prevented it from being developed as a selection tool for generating transgenic parasites in these systems. The development of new selectable markers for rodent parasites might negate this problem and allow a similar expression system to be developed.

Other systems have been described recently that allow the conditional activation of a transgene[11], however these systems are more complex and hence more difficult to establish. For instance, the Atc-regulatable system[8] requires two different components that either need to be inserted in two different selection cycles or are provided on one very large construct. The utility of the FKBP12 system[9] is dependent on the confirmation of the protein of interest fused to the protein destabilization domain and the efficacy of the system might vary largely between different proteins investigated. The system described here is not an inducible system and there is no possibility to switch it off completely. It rather provides the possibility to adjust the level of gene expression by selection for transgene copy number. Therefore, gene regulation is not possible within hours but, depending on the desired expression level, up to 2–3 weeks of selection are needed. The significant advantage is the simplicity and hence stability of the bidirectional constructs. Manipulation by DNA cloning is relatively easy and the initial generation of stable transformants is straightforward. During cloning of the several constructs described here not a single deletion or truncation event was observed. While the use of the same 3' UTR sequence for both transgenes could potentially lead to deletion of the intervening sequence during propagation in *E. coli*, such deletions were never detected in any of our small or large scale plasmid DNA preparations, suggesting that the constructs are quite stable. In addition, the identical 3'UTR sequences are oriented in opposite directions and therefore homologous recombination would be expected to produce an inversion of the entire expression cassette rather than a deletion.

## Conclusion

The ability to genetically manipulate cultured malaria parasites has made a tremendous impact on the understanding of the molecular biology of these organisms. As knowledge of these pathogens becomes more advanced, the ability to perform more precisely regulated experiments will be needed. By using the adjustable transgene expression system described here, it should be possible to control the expression level of a gene of interest, thus allowing a researcher to determine the effect of normal expression, under-expression or over-expression on parasite growth, metabolism or viability. This type of "fine-tuning" of expression levels will be particularly important for the study of protein localization, where mis-timing or over-expression can lead to incorrect localization of a protein. Similarly, manipulating the level of transgene expression can be useful in complementation studies where the chromosomal copy of a gene has been knocked out, or in cases where expression of a dominant negative version of a protein requires particularly high expression levels to cause a phenotype. For dominant negatives, the expression level of the transgene could potentially be adjusted to a level that displays a phenotype but remains sub-lethal. The ability to control transgene expression therefore has added an additional tool to the repertoire of molecular techniques and provides a relatively simple and robust alternative for the specific analysis of gene function in *Plasmodium falciparum*.

## Authors' contributions

CE and DR conducted the experiments and collected the data. CE and KWD designed experiments and analysed the data. CE and KWD wrote the paper.
